# Circular RNA Eps15-homology domain-containing protein 2 induce resistance of renal cell carcinoma to sunitinib via microRNA-4731-5p/ABCF2 axis

**DOI:** 10.1080/21655979.2022.2059960

**Published:** 2022-04-12

**Authors:** Wen Li, GaiXia Li, LuQuan Cao

**Affiliations:** a Department of Laboratory, Affiliated Hospital of Shandong University of Traditional Chinese Medicine, Jinan City, Shandong Province, China; b Department of Electrocardiography Room, Qingdao Women’s and Children’s Hospital, Qingdao City, Shandong Province, China; c Department of Prenatal Diagnosis Center, Jinan Maternal and Child Health Hospital Prenatal Diagnosis Center, Jinan City, Shandong Province, China

**Keywords:** ABCF2, circular RNA eps15-homology domain-containing protein 2, drug resistance, MicroRNA-4731-5p, renal cell carcinoma, sunitinib

## Abstract

Circular RNAs (circRNAs) are linked with the occurrence and progression of renal cell carcinoma (RCC). However, circRNAs’ mechanism in developing resistance to RCC has not been clarified. This research assessed the role and mechanism of circular RNA circ Eps15-homology domain-containing protein 2 (EHD2) in the resistance of sunitinib (SU) to RCC. ACHN, 786-O, 769P, and HEK-293 T cells and RCC tissue samples were used for the investigations. The circEHD2 expression in RCC cells and tissues was determined through RT-qPCR. Association of circEHD2 with RCC histological grade of RCC was done through Chi-square. MiR-4731-5p, ABCF2, and circEHD2 were transfected into RCC cell lines. A dual-luciferase reporter assay was used to determine the interaction between miR-4731-5p, circEHD2, and ABCF2. MTT assay was used to analyze cell viability, while apoptosis was studied using flow cytometry. Colony-formation and transwell experiments were used to assess migration and invasion. The ATP Binding Cassette Subfamily F Member 2 (ABCF2) expression was analyzed through western blot. The results showed increased circEHD2 in SU-resistant RCC tissues and cell lines and implicated in RCC histological grade and SU resistance. Knock-down of circEHD2 down-regulated the resistance of RCC to SU *in vitro* and *vivo*; circEHD2 bound to miR-4731-5p to mediate ABCF2 in RCC; ABCF2 rescued the inhibitory effect of circEHD2 knock-down on SU resistance of RCC. In conclusion, circEHD2 enhances RCC resistance to SU via acting as a miR-4731-5p sponge to mediate ABCF2. MiR-4731-5p can target circEHD2 and ABCF2, thus providing a novel and effective therapeutic against renal cell carcinoma.

## Graphical Abstract

**Figure:**
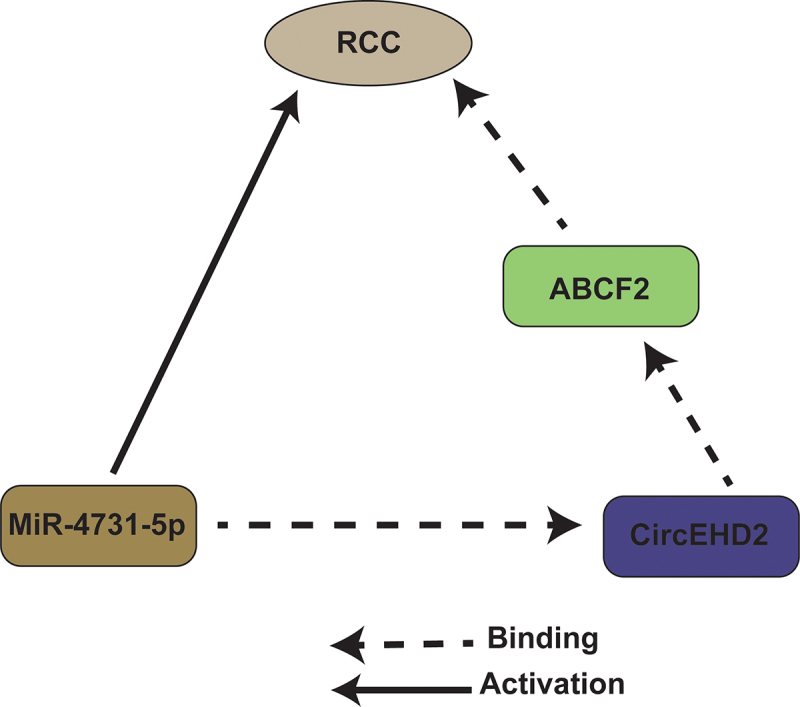

## 1. Introduction

Renal cell carcinoma (RCC) is a highly lethal urinary system tumor with a rising global incidence [1]. RCC is broadly classified into clear cell renal cell carcinoma, chromophobe RCC and multilocular cystic renal neoplasm of low malignant potential. Among these, clear cell renal cell carcinoma forms 70% of all cases, occurs sporadically in almost 95% [2] of the cases and most drugs develop resistance to this subtype. The current treatment methods for RCC include surgical resection and drug therapy. Nevertheless, drug therapy reduces the outcome due to emergence of resistance [3], while surgery is only suitable for early RCC [4]. Approximately one-third of RCC patients are only diagnosed during the manifestation of locally advanced or metastatic tumors, thus greatly reducing their overall survival rate [5]. Angiogenesis has presently been reported as vital to the progression of malignant RCC. Consequently, various angiogenesis inhibitors have been applied in preclinical and clinical trials, and anti-angiogenesis use has become among the options of drug therapy for RCC [6].

Sunitinib (SU) is a tyrosine kinase inhibitor targeting various tumor proliferation and angiogenesis receptors, such as vascular endothelial growth factor receptor and platelet-derived growth factor receptor [7]. SU has been approved for treating gastrointestinal stromal tumors and advanced RCC [8]. As a first-line therapeutic drug for metastatic or advanced RCC, SU has significantly enhanced RCC prognosis among patients [9]. Nevertheless, resistance to SU has been recently reported, thus reducing RCC outcomes [10]. Consequently, understanding the latent mechanism involved in resistance would be critical in developing possible predictive biomarkers for RCC.

Circular RNA (circRNA) is an endogenous non-coding RNA originating from the gene’s exon or intron region. CircRNA is more stable and less liable to degrade via RNase R treatment than linear RNA [11]. Several new circRNAs have recently been found in various cell types [12]. According to previous studies, circRNAs play important roles in various pathophysiological processes and enhance cancer progression [13]. Meanwhile, recent research reported that circRNA controls gene expression via competitive binding with specific microRNAs (miRNAs) [14]. For instance, circRNA cRAPGEF5 represses the growth and metastasis of RCC via the miR-27a-3p/Thioredoxin-interacting protein pathway [15], whileCircPCNXL2 binding with miR-153 elevates renal cancer cell progression via up-regulating Zinc Finger E-Box Binding Homeobox 2 [16]. A previous study also reported that circRNA Eps15-homology domain-containing protein 2 (EHD2) is induced in RCC and can be used as a diagnostic and prognostic biomarker for RCC [17]. Nevertheless, the role of circEHD2 in RCC resistance to SU has not been elucidated.

The current study hypothesized that circEHD2 induces renal cell carcinoma resistance to sunitinib via microRNA-4731-5p/ABCF2 axis. The study aimed to determine circEHD2 expression in SU-resistant RCC tissues and cell lines, to understand the effect of circEHD2 inhibition on SU-resistance to RCC, to determine whether circEHD2 and ABCF2 are targets of miR-4731-5p in the inhibition of RCC, and to understand the effect of ABCF2 on circEHD2 in SU-resistance to RCC.

## 2. Materials and methods

### 2.1 Ethical statement and tissue samples

The investigation was approved by the Ethics Committee of the Affiliated Hospital of Shandong University of Traditional Chinese Medicine (EC/ACS/102-99B). Written informed consent was obtained from all the study participants. RCC positive human tissue samples were obtained from 107 patients, while adjacent normal tissues (≥ 5 cm from the edge of the tumor) were obtained from 79 patients admitted to the Affiliated Hospital of Shandong University of Traditional Chinese Medicine. All the RCC positive samples showed clear histopathological diagnosis features.. RCC patients were treated with SU chemotherapy every 3 weeks for 4 cycles, as recommended by the National Comprehensive Cancer Network guidelines. After 3 months of chemotherapy, tumor shrinkage of over 30% or disappearance was judged as non-drug resistance, while tumor enlargement of over 30% or the appearance of new metastases was determined as drug resistance.

### 2.2 Cell culture and establishment of SU-resistant cell lines

The RCC cell lines (769P, 786-O, and ACHN) and human embryonic kidney cell line (HEK-293 T) were purchased from the American Type Culture Collection (Manassas, Virginia, USA). The cells were cultured in Dulbecco’s modified Eagle’s medium (DMEM) (Invitrogen, Carlsbad, CA, USA) supplemented with 10% Fetal Bovine Serum (FBS, Thermo Fisher Scientific, MA, USA). To get a drug-resistant 786-O-R cell, the parental 786-O cells were treated with SU Malate (Selleck Chemicals, Houston, TX, US) at an initial dose of 0.25 µM. Subsequently, the concentration of SU was increased by 0.25 µM every 72 h. the surviving clones were continuously passaged until the IC50 values reached 18 µM in the 10^th^ month. The stable SU-resistant sublines were maintained with the medium supplemented by 0.02 µM SU [18].

### 2.3 RNase R treatment

The RCC cells were treated with RNase R (2 U/µg, GeneSeed, Guangzhou, China) and incubated and inactivated. Reverse transcription of the treated RNAse was done using divergent primers or convergent primers, then detected using reverse transcription-quantitative polymerase chain reaction (RT-qPCR). The experiment was repeated three times, and the data were averaged.

### 2.4 Cell transfection

Approximately 2 × 10^5^ cells were seeded per well in a 6-well plate and cultured to a confluence of 60%. The cells were then incubated with a serum-free culture medium at 37°C. Later, the cells were transfected with 50 nM si-negative control (NC)/circEHD2#1/circEHD2#2, mimic NC, miR-4731-5p mimic, and pcDNA-NC/ABCF2 plasmids using Lipofectamine 2000 transfection reagent (Invitrogen, USA) according to the manufacturer’s instructions. All the plasmids and primers were purchased from GenePharma Co. Ltd. (Shanghai, China).

### 2.5 Reverse transcription-quantitative polymerase chain reaction (RT-qPCR)

Extraction of total RNA from cells and tissues was done using Trizol (Invitrogen, USA). Determination of the RNA concentration) was done using Nanodrop 2000 (Thermo Fisher Scientific). The total RNA (1 mg) was reverse-transcribed into complementary DNA using the PrimeScript RT kit (Takara Holdings, Kyoto, Japan). SYBR Premix Ex Taq (Tli RNaseH Plus) kit (Takara Holdings, Kyoto, Japan) was used, and real-time PCR was carried out on an ABI7500 qPCR instrument (Thermo Fisher Scientific, USA). U6 and GAPDH were used as endogenous controls to normalize gene expression. The relative gene expression was determined through 2^−ΔΔCt^. The primers used were listed in Table 1.

**Table 1.** Primer sequence

Genes	Primer sequence (5’-3’)
MiR-4731-5p	F: TGCTGGGGGCCACAT
	R: CTCTACAGCTATATTGCCAGCCAC
U6	F: CTCGCTTCGGCAGCACA
	R: AACGCTTCACGAATTTGCGT
CircEHD2	F: CTGGTGCGAGCTACGACTTC
	R: TCGTCCGAGATCTCCAGCTT
ABCF2	F: GAGGTTTCACTGGGAGCAAGATC
	R: CTGTAGCGTCTTCTCCTTGCTC
GAPDH	F: GCTCTCTGCTCCTCCTGTTC
	R: ACGACCAAATCCGTTGACTC

### 2.6 Western blot

After transfection, the total protein was extracted from cells and tissues using the Radio-Immunoprecipitation assay lysis buffer. A bicinchoninic acid protein assay kit (Thermo Fisher Scientific, USA) was used to measure protein concentration. The protein was separated using 10% sulfate-polyacrylamide gel electrophoresis, and the electro-blot was transferred onto a Polyvinylidene fluoride membrane. The membrane was then blocked with 5% skimmed milk and incubated with primary antibodies ABCF2 (1: 1000), GAPDH (1: 1000) ((Abcam, Cambridge, MA, USA) at 4 C overnight and washed. The membrane was then incubated with corresponding secondary antibodies (Shanghai Miaotong Biotechnology Co., Ltd., Xuhui District, Shanghai, China) for 1 h at room temperature. The band signal was detected using enhanced chemiluminescence reagent in a Bio-rad Gel Dol EZ imager (Gel DOC EZ IMAGER, Bio-rad, California, USA). Image J software was used for quantifying the intensity of each band. The relative expression of the target protein was standardized using GAPDH.

### 2.7 The luciferase activity assay

The online bioinformatics tools miRanda (www.microrna.org), PicTar (pictar.mdc-berlin.de/), and TargetScan (www.targetscan.org) were used in predicting the binding site of circEHD2/ABCF2 with miR-4731-5p. The luciferase activity assay was used to verify the binding/targeting link of circEHD2/ABCF2 with miR-4731-5p. The gene synthesis technology was used to synthesize DNA fragments covering this site and mutants of this site. The pGL3 reporter vector (Promega, Wisconsin, USA) was used for the construction of wild/mutant-type circEHD2/ABCF2 3’-untranslated region (UTR) (circEHD2/ABCF2-WT/MUT) plasmids. cells in good growth condition at the logarithmic growth phase were collected and adjusted to a density of 2 × 10^5^cells/mL, and cultured with DMEM supplemented with 10% FBS. The cells were co-transfected with circEHD2/ABCF2-WT/MUT and mimic control/miR-4731-5p mimic genes, using Lipofectamine2000 transfection reagent ((Invitrogen, USA) following the manufacturer’s instructions. The cells were then cultured for 48 h. The luciferase activity was later determined using the Dual-Luciferase Reporter Assay System (Promega). All the experiments were done in triplicates.

### 2.8 RNA-pull down

The pull-down assay was carried out as previously described [19]. The biotin-labeled miR-4731-3p WT or biotin-labeled miR-4731-3p MUT plasmids (50 nM each) were separately transfected into the RCC cells. The cells were then collected and incubated with a specific cell lysate (Ambion, Austin, Texas, USA). Then, 50 mL of sample cell lysate was loaded. The remaining lysate was incubated with M-280 streptavidin magnetic beads (Sigma, St Louis, MO, USA) pre-coated with RNase-free and yeast tRNA (Sigma). Samples were then washed with cold lysis buffer and low/high-salt buffer. An antagonistic miR-4731-5p probe was set up as a negative control. After extraction of total RNA via Trizol, the RT-qPCR was used to detect circEHD2. The experiment was repeated three times, and the data were averaged.

### 2.9 3-(4, 5-dimethylthiazol-2-yl)-2, 5-diphenyltetrazolium bromide (MTT)

The cells in each group were homogenized, and approximately 1 × 10^4^cells/mL were seeded per well in a 96-well plate. Cells were then cultured for 24, 48, and 72 h, and 5 mg/mL (20 μL) MTT reagent was added to each well and incubated for 4 h. The supernatant was aspirated from the culture wells, 150 μL dimethyl sulfoxide was added to each well, and the culture plate was gently shaken. The optical density value (OD490) at 490 nm wavelength of each well was determined using a microplate reader (Bio-tekELx800, US). The experiment was done in triplicates.

### 2.10 Plate colony formation

The cells from each group were harvested at the logarithmic growth phase, trypsinized, serially diluted, and counted under the microscope. Approximately 200 cells/ well were seeded in a 6-well plate and incubated for 9 days. The medium was discarded, and cells were fixed using 4% paraformaldehyde. The fixative was then removed, and cells were stained for 1 h at room temperature using crystal violet. The cells were then washed, colony formation was determined under a fluorescent microscope, and colony numbers counted. The percentage rate of colony formation was calculated as colony number per 2 × 10^2^ cells × 100, as described elsewhere [20]. The experiment was done in triplicates.

### 2.11 AnnexinV-fluorescein isothiocyanate (FITC)/propidium iodide (PI)

The cells (2. 0 × 10^4^ per well) in the logarithmic growth phase were seeded in a 6-well culture plate. Later, the cells were detached with 0.25% trypsin (without ethylene diamine tetraacetic acid), collected in a flow tube, centrifuged, and the supernatant was finally discarded. The cells were then incubated with 500 μL loading buffer, 5 μL Annexin V-FITC, and 10 μL PI solution as stipulated in Annexin-V-FITC Cell Apoptosis Detection Kit (Biovision, K101). The apoptosis rate was determined in a flow cytometer (BD Biosciences, Franklin Lakes, NJ, USA). The experiment was repeated three times, and the data were averaged.

### 2.12 Transwell

Transwell experiment was used to determine cell migration and invasion, as reported previously [21]. A chamber of 8 μm pores (6.5 mm) (Corning Costar Corp., USA) was used in the experiment. To study cell migration, 2 × 10^4^ stably transfected cells were resuspended in 200 μl of serum-free DMEM and plated in the upper chamber. Later, the media (500 μl) supplemented with 10% fetal bovine serum was introduced in the lower well chamber. Cells were then incubated under the chemotactic condition at 37°C for 24 h. The cells were stained for 30 min using 1% crystal violet, and cells on the membrane’s upper surface were removed using cotton swabs. The cells on the bottom of the membrane were quantified and imaged microscopically (Olympus Corp. Tokyo, Japan) in four random fields. For the invasion assay, Matrigel, 0.1 ml (50 μg/ml, BD Biosciences, USA) was added to the plate and incubated for 2 h. The remaining assay steps were similar to the migration assay described above. The experiments were done in triplicate.

### 2.13 Statistical analysis

Data statistical analysis was done using GraphPad Prism 8. The data were presented as mean ± standard deviation. A two-group comparison was done using a t-test, while the comparison among multiple groups was done with a one-way analysis of variance (ANOVA). After ANOVA analysis, Tukey’s multiple comparisons test was used for the pairwise comparison. Analysis of the association between circEHD2 and the clinicopathological features of RCC patients was done via the chi-square test. *P* < 0.05 was considered statistically significant.

## 3. Results

### 3.1 CircEHD2 is elevated in SU-resistant RCC tissues and cell lines

Renal cell carcinoma (RCC) is a highly prevalent and fatal urinary system tumor. Circular RNAs (circRNAs) are linked with the occurrence and progression of renal cell carcinoma (RCC). The study hypothesized that circEHD2 induces renal cell carcinoma resistance to sunitinib via microRNA-4731-5p/ABCF2 axis. The study aimed to determine circEHD2 expression in SU-resistant RCC tissues and cell lines, to understand the effect of circEHD2 inhibition on SU-resistance to RCC, to determine whether circEHD2 and ABCF2 are targets of miR-4731-5p in the inhibition of RCC, and to understand the effect of ABCF2 on circEHD2 in SU-resistance to RCC.

**Figure 1.**
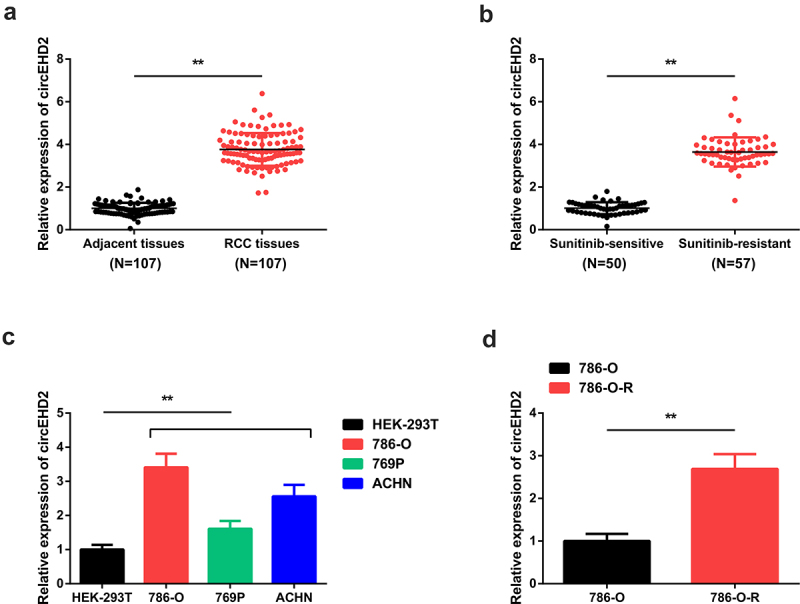
CircEHD2 is elevated in SU-resistant RCC tissues and cell lines. A: Assessment of circEHD2 mRNA expression in adjacent and RCC tissues using RT-qPCR; B: determination of circEHD2 mRNA expression in su-sensitive and su-resistant tissues using RT-qPCR; C: Examination of circEHD2 expression in RCC cell lines done through RT-qPCR; D: Detection of CircEHD2 mRNA expression in parental strain 786-O and the drug-resistant strain 786-O-R cell lines using RT-qPCR. *P < 0.05, **P < 0.01.

To determine the role of circEHD2 in RCC, the mRNA expression of circEHD2 in RCC cells and tissues was analyzed through RT-qPCR. According to the results, circEHD2 mRNA expression was significantly elevated in SU-resistant RCC tissues compared to the adjacent normal tissues (Figure 1a). Further, circEHD2 mRNA expression was significantly increased in SU-resistant tissues compared to the SU-sensitive tissues (Figure 1b). The circEHD2 mRNA expression was also significantly increased in 786-O, 769P, and ACHN cells compared to the human embryonic kidney cell line (HEK-293 T) (Figure 1c). Finally, circEHD2 was significantly increased SU-resistant strain 786-OR cells compared to the parental strain 786-O cells (Figure 1d). Chi-square test analysis of the link of circEHD2 with clinicopathological parameters of RCC patients indicated that increased circEHD2 was implicated in histological grade and SU resistance (Table 2).

**Table 2.** Enhanced circEHD2 was implicated in histological grade and SU resistance

Clinicopathologic data	n	CircEHD2		*P*
	107	Reduction group (n = 53)	Elevation group (n = 54)	
Age (years)				0.382
< 60	67	31	36	
≥ 60	40	22	18	
Gender				0.633
Male	71	34	37	
Female	36	19	17	
Histologic grade				0.002
1–2	39	27	12	
3–4	68	26	42	
Resistance to SU				0.005
Sensitive		32	18	
Resistant		21	36	

### 3.2 CircEHD2 inhibition reduces the resistance of RCC to SU

**Figure 2.**
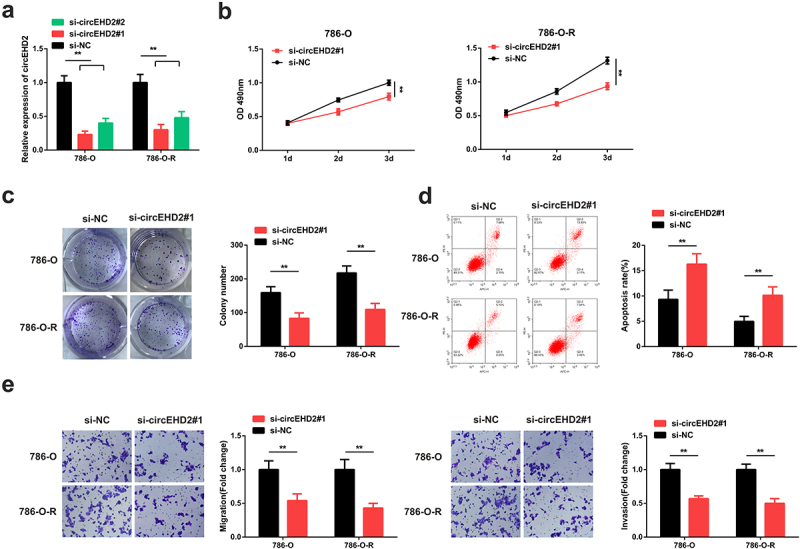
CircEHD2 inhibition reduces the resistance of RCC to SU. A: RT-qPCR detection of circEHD2 mRNA expression in parental strain 786-O and the drug-resistant strain 786-O-R cell lines cells after transfection with si-circEHD2#2, si-circEHD2#1 or si-NC plasmids; B: detection of cell proliferation in the transfected cells using MTT method; C: Colony formation assay for the detection of colony formation in si-circEHD2#1 transfected cells; D: detection of cell apoptosis using annexinv-FITC/PI and flow cytometry; E: Transwell detection of cell migration and invasion following the transfection of the 786-O-R and 786-O cells; *P < 0.05, **P < 0.01. N = 3.

To investigate the role and mechanism of circEHD2 in RCC resistance to SU, si-circEHD2#1 and circEHD2#2 were separately transfected into 786-O and 786-OR cells. To verify the transfection efficiency, circEHD2 expression was determined through RT-qPCR. The results confirmed a significantly suppressed circEHD2 expression in both the cell lines (Figure 2a). Meanwhile, the knock-down effect of si-circEHD2#1 was better compared to circEHD2#2, so the selection of the cells transfected with si-circEHD2#1 was used for subsequent functional experiments. Cell proliferation was then determined in the transfected cells through MTT experiments. The MTT results confirmed a significantly reduced cell growth in the si-circEHD2#1-transfected cells compared to the si-NC (Figure 2b). The colony formation assays also demonstrated significantly reduced colony numbers in both the cells transfected with circEHD2#1 compared to the si-NC (Figure 2c).

Further, apoptosis was investigated through the flow cytometry assay. The results confirmed significantly elevated apoptosis in the 786-O and 786-O-R cells after transfection with circEHD2#1 compared to the si-NC (Figure 2d). Finally, the cell migration and invasion done through transwell experiments confirmed a significant reduction of migration and invasion following transfection with circEHD2#1 compared to the si-NC (Figure 2e). These data hence confirmed that the suppression of cicEHD2 inhibits the resistance of RCC to SU.

### 3.3 CircEHD2 and ABCF2 are targets of miR-4731-5p in the inhibition of RCC

**Figure 3.**
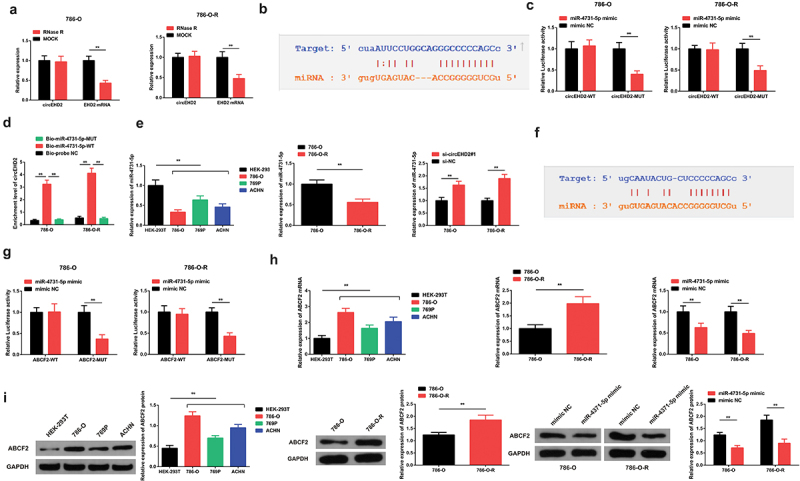
CircEHD2 and ABCF2 are targets of miR-4731-5p in the inhibition of RCC. A: RNase R treatment confirmation of the circular features of circEHD2; B, F: prediction of the binding site of circEHD2 and miR-4731-5p using the online bioinformatics tools; C: The luciferase activity assay of circEHD2 and miR-4731-5p, and the targeting link between miR-4731-5p and circEHD2; D: RNA pull-down detection of the enrichment of circEHD2 by miR-4731-5p; E: RT-qPCR detection of miR-4731-5p in 786-O, 769P, ACHN, and HEK-293 T cells; F: assessment of the binding of miR-4731-5p and ABCF2, G; The luciferase activity and the targeting link between miR-4731-5p and ABCF2, H: RT-qPCR detection of ABCF2 in cells. I: WB detection of ABCF2 in cells * *P* < 0.05; **P < 0.01. N = 3.

Verification of the stability of circEHD2 confirmed that circEHD2 exhibited increased tolerance to RNase R treatment compared to linear EHD2 following the exposure to RNase R (Figure 3a). We hypothesized that circEHD2 functioned as a competing endogenous RNA modulating the SU resistance of RCC. To confirm our theory, online bioinformatics tools were used to predict the binding sites of circEHD2. According to the results, circEHD2 binds with miR-4731-5p at the 3ʹUTR region (Figure 3b). The luciferase activity was determined using the dual-luciferase reporter assay. Our observations confirmed that the luciferase activity of WT circEHD2 + miR-4731-5p mimic was significantly reduced compared to the mimic-NC. However, the luciferase activity in the MUT circEHD2 + miR-4731-5p mimic one was not changed, confirming that circEHD2 binds with miR-4731-5p (Figure 3c). Additionally, the enrichment of circEHD2 in the Bio-miR-4731-5p-WT was significantly elevated compared to the Bio-probe NC, but there was no observable change in Bio-miR-4731-5p–MUT group (Figure 3d). Together, these results confirmed that circEHD2 binds with miR-4731-5p. Further, the expression of miR-4731-5p was assessed in the 786-O-R and 786-O cells. The results confirmed a significantly down-regulated miR-4731-5p expression in the 786-O-R and 786-O cells RCC cell lines compared to HEK-293 T cells (Figure 3e). However, the miR-4731-5p mRNA expression was significantly increased following the transfection of 786-O-R and 786-O cells with si-circEHD2#1 compared with the si-NC plasmids (Figure 3e).

Further, the possible binding of miR-4731-5p with ABCF2 was predicted using bioinformatics tools. The results confirmed that miR-4731-5p binds to ABCF2 at the 3ʹUTR region (Figure 3f). To confirm the targeting link of miR-4731-5p and ABCF2, the mutant sequence and wild sequence of ABCF2 3ʹUTR missing miR-4731-5p binding site were simultaneously designed and inserted into the reporter plasmid. The dual-luciferase reporter assay was then used to determine the luciferase activity. The results indicated an insignificant change in the luciferase activity in the miR-4731-5p mimic of the MUT-miR-4731-5p/ABCF2 plasmid group, but a significantly reduced activity in the WT–miR-4731-5p/ABCF2 plasmid group (Figure 3g).

The relative ABCF2 mRNA expression was significantly increased in the 786-O, 769P, and ACHN cells compared to the HEK-293 T cells. Further, ABCF2 mRNA expression was significantly elevated in the 786-O-R compared to the 786-O group. However, the ABCF2 mRNA expression was significantly reduced in the miR-4731-5p mimic compared to the mimic-NC group in the 786-O and 786-O-R cell lines (Figure 3h). Finally, the ABCF2 protein expression was assessed through western blotting. The results confirmed a significant increment of ABCF2 in the 786-O, 769P, and ACHN cells compared to HEK-293 T cells. ABCF2 was also elevated in 786-O-R compared to the 786-O group. However, the ABCF2 protein was significantly reduced in the miR-4731-5p mimic transfected compared to the mimic-NC group (Figure 3i). These results confirmed that miR-4731-5p targeted ABCF2, thereby impacting it.

### 3.4 ABCF2 reverses the inhibitor effect of circEHD2 on SU resistance of RCC

**Figure 4.**
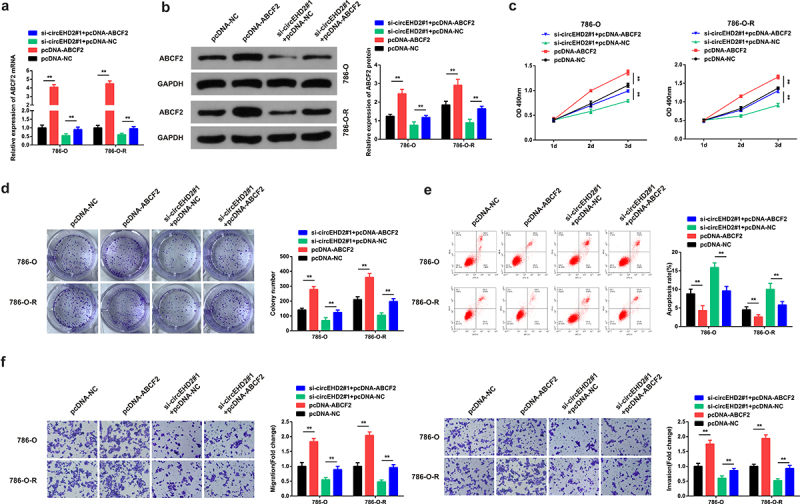
ABCF2 reverses the inhibitor effect of circEHD2 on SU resistance of RCC. A, B: RT-qPCR and WB detection of circEHD2 in cells after transfection with si-circEHD2#1+ pcDNA-ABCF2, si-circEHD2#1+ pcDNA-NC; pcDNA-ABCF2 or pcDNA-NC C: MTT method for detection of cell proliferation; D: Colony formation assay detection of colony formation in cells after transfection; E: Annexinv-FITC/PI detection of cell apoptosis rate; F: Transwell detection of cell migration and invasion; *P < 0.05, **P < 0.01. N = 3.

The role of ABCF2 in modulating SU resistance by circEHD2 was also investigated. To understand this, the si-circEHD2#1+ pcDNA-ABCF2, si-circEHD2#1+ pcDNA-NC, pcDNA-ABCF2, or pcDNA-NC plasmids were transfected into 786-O and 786-OR cells. ABCF2 mRNA and protein expression was then determined using the RT-qPCR and western blotting.. The results confirmed significantly reduced ABCF2 mRNA and protein expression in the si-circEHD2#1+ pcDNA-ABCF2 compared to the pcDNA-ABCF2 group in both the 786-O and 786-O-R cells (Figure 4a-b). The cell viability experiments through MTT demonstrated significantly increased cell proliferation in the pcDNA-ABCF2 group compared to the si-circEHD2#1+ pcDNA-ABCF2 in both 786-O and 786-OR cells (Figure 4c).

The colony formation assay confirmed significantly reduced colony numbers in the si-circEHD2#1+ pcDNA-ABCF2 compared to the pcDNA-ABCF2 groups (Figure 4d). Apoptosis was also investigated using the flow cytometry experiment, and the observations confirmed significantly increased apoptosis in the si-circEHD2#1+ pcDNA-ABCF2 compared to pcDNA-ABCF2 groups (Figure 4e). The use of transwell assays to determine the migration and invasion ability demonstrated a significant decrease in the migration and invasion ability following transfection with si-circEHD2#1+ pcDNA-ABCF2 plasmids compared to pcDNA-ABCF2 in both the cells (Figure 4f). In summary, these results confirmed that ABCF2 turns around the inhibitory role of circEHD2 in the resistance of SU to RCC.

## 4. Discussion

RCC remains fatal urinary system cancer, with tyrosine kinase inhibitor (TKI) being the preferred cure for metastatic RCC (mRCC) [22]. SU is a popular tyrosine kinase inhibitor that provides a promising outcome against RCC in the initial stages. However, patients later develop resistance to this treatment. Additionally, the long-term application of SU therapy elevates lysosomal biosynthesis and exocytosis, thus triggering metastasis of RCC [23]. Consequently, further understanding of RCC pathogenesis and the molecular mechanism of resistance to SU could help develop new biomarkers to prevent SU resistance. In this research, circEHD2 was elevated in SU-resistant cell lines and tissues, and its upregulation was linked with histological grade and SU resistance. The study clarified that inhibition of circEHD2 restrained SU resistance to RCC. The investigation also reported that circEHD2 suppression down-regulated resistance of RCC to SU via restraining ABCF2 through miR-4731-5p targeting. Hence, it was confirmed that circEHD2 plays an essential function in SU resistance to RCC via miR-4731-5p binding to mediate ABCF2.

CircRNA is a transcription product, and its dysregulation leads to tumorigenesis [24]. According to a recent study, RCC-specific circRNA expression profiles including circEGLN3, circNOX4, circRHOBTB3 [25], circEHD2, circENGLN3, and circNETO2 are used to distinguish tumors from normal kidney tissues [17]. Among them, circEHD2 is significantly up-regulated and considered a promising RCC biomarker. Elevated circEHD2 is an independent predictor of progression-free and cancer-specific survival in patients experiencing radical or partial nephrectomy [17]. Nevertheless, the role and mechanisms of circEHD2 in RCC remains unclear. The current research confirms that circEHD2 is up-regulated in RCC and is implicated in SU resistance and progression of RCC. It was confirmed that circEHD2 knock-down reduced SU-resistant cancer cells’ progression. Our study is in agreement with a previous investigation which confirmed that EHD2 elevation enhances RCC proliferation, migration and invasion [26]. Several studies have confirmed that circRNA has multiple sites to bind and control miRNA activity and modulate downstream target genes. This mechanism has been confirmed in various cancers, including RCC [27]. Hence, in this research, the downstream target genes of circEHD2 was further ascertained, clarifying that circEHD2 bind with miR-4731-5p in RCC.

Multiple studies have reported that miRNAs are important in the pathogenesis and useful diagnostic, prognostic, and predictive biomarkers for various cancers such as RCC [28]. For instance, miR-23a-3p is up-regulated in RCC tissues and cell lines and can be a prognostic biomarker for RCC [29]. MiR-1246 controls the progression of RCC via targeting CXCR4 [30]. MiR-4731-5p is dysregulated and plays various roles in various tumors such as nasopharyngeal carcinoma [31], lung adenocarcinoma [32], and liver cancer [33]. Moreover, miR-4731-5p has also been implicated in several biological activities such as cell proliferation and migration [34]. Nevertheless, no research has been done to determine the role of miR-4731-5p on RCC. We report that miR-4731-5p has a repressive function in RCC and reduces SU resistance via targeting ABCF2.

ABCF2 is a member of the ABCF transporter family, a subgroup of the ATP binding cassette transporter superfamily, and is linked with drug resistance in several cancers [35]. For instance, ABCF2 is a Nrf2 target gene contributing to cisplatin resistance in ovarian cancer cells [36]. ABCF2 may function in tumor suppression at the metastatic site of breast cancer (BC) and the endocrine pathway of BC and be implicated in BC’s chemotherapy resistance [37]. This investigation confirmed that ABCF2 was up-regulated in RCC cells. Increased ABCF2 reversed the impact of circEHD2 knock-down and facilitated 786-O and 786-OR cell growth. The current work is the first study on ABCF2 in RCC, as far as we know. The above results clarified that circEHD2/miR-4731-5p/ABCF2 axis plays a vital role in RCC SU resistance. The limitation of this study is in the lack of data to establish a clear relationship between ABCF2 and circEHD2 animal model experiments to provide more information and justification of our data. The future perspective may focus on establishing RCC animal model that can be used to study the mechanisms and control of SU-resistance to RCC.

## 5. Conclusion

In conclusion, circNOLC1 is increased during the progression of renal cell carcinoma; CircEHD2 inhibition reduces the resistance of RCC to SU; miR-4731-5p directly targets the circEHD2 and ABCF2 to arrest the establishment of renal cell carcinoma, and ABCF2 reverses the inhibitor effect of circEHD2 on SU resistance of RCC. The use of miR-4731-5p to target circEHD2 and ABCF2 might provide a novel and effective therapeutic against renal cell carcinoma.
